# Lymph node metastasis as the initial symptom of a germ cell tumor in an adult: A case report

**DOI:** 10.1097/MD.0000000000029281

**Published:** 2022-07-29

**Authors:** Fang Guo, Hongbing Wang, Heshun Xia, Hongwei Shi, Peng Xu, Guoliang Pi

**Affiliations:** a Department of Pathology, Hubei Cancer Hospital, Tongji Medical College, Huazhong University of Science and Technology, Wuhan, Hubei, China; b Department of Gynecology and Oncology, Hubei Cancer Hospital, Tongji Medical College, Huazhong University of Science and Technology, Wuhan, Hubei, China; c Department of Radiation Oncology, Hubei Cancer Hospital, Tongji Medical College, Huazhong University of Science and Technology, Wuhan, Hubei, China; d Department of Digestive and Respiratory, Shiyan Hospital of Traditional Chinese Medicine, Shiyan, Hubei, China.

**Keywords:** chemotherapy, germ cell tumor, immature teratoma, lymph node metastasis

## Abstract

Germ cell tumors in the head and neck are very rare. In cases of germ cell tumors, it is uncommon for lymph node metastasis to be the only and initial symptom, and this can easily lead to a misdiagnosis. Herein, we report about a 28-year-old woman with lymph node metastasis, in whom a primary tumor appeared in the nasal cavity.

**Patient concerns:**

A 28-year-old woman presented with enlarged left submandibular lymph nodes. No other mass was found on whole-body screening using positron emission tomography-computed tomography.

**Diagnosis:**

After partial submandibular lymphadenectomy was performed, histopathological and immunohistochemical examinations revealed a metastatic germ cell tumor. However, it was difficult to further classify and affirm the origin.

**Interventions:**

As the patient was receiving four cycles of bleomycin, etoposide, and cisplatin chemotherapy, a primary tumor emerged in the nasal cavity, which was finally confirmed as an immature teratoma of a high World Health Organization histological grade and Norris grade 3. This tumor was found to contain similar components to lymph nodes with respect to histopathological and immunohistochemical characteristics, especially the immature neural tubes or nervous tissue in the nasal cavity. Fortunately, the patient recovered well with no signs of relapse, and the size of residual lymph nodes remained unchanged after she received another four cycles of bleomycin, etoposide, and cisplatin chemotherapy and two cycles of doxorubicin and ifosfamide (AI) chemotherapy.

**Outcomes:**

Unfortunately, 11 months later, during the coronavirus disease pandemic, the patient died owing to respiratory failure and pulmonary infection.

**Conclusions:**

In cases of malignant tumor in the submandibular lymph nodes of adults, the metastasis of a germ cell tumor should be considered an important differential diagnosis even if a primary tumor does not emerge. In this case, adequate postoperative chemotherapy is necessary.

## 1. Introduction

Germ cell tumors rarely appear in the head and neck, and such cases account for only 6% of germ cell tumor cases.^[1]^ Reported cases usually involve teratomas with immature or mature somatic type tissues^[2]^ or yolk sac tumors^[3]^ found in different sites, including the soft tissues of the head and neck region, nasal cavity,^[4]^ anterior region of the neck, forehead, orbit, floor of the mouth, nasopharynx, and oropharynx.^[5]^ Distinguishing between immature and mature tissues is usually straightforward, and it is not difficult for a pathologist to make a correct diagnosis by identifying the multiple components in a primary tumor. However, in some cases, when a primary tumor is absent or concealed, even if positron emission tomography (PET) shows multiple lymph nodes enlarged and indicates metastasis, formulating a correct pathologic diagnosis by biopsy can be particularly challenging because the malignant component in metastases might be a non-specific part of the primary tumor. In such cases, in order for the pathologists to formulate an accurate diagnosis, they have to carefully identify a variety of probable tumor types and successively rule out different possibilities. Furthermore, if the possibility of a germ cell tumor is ignored or overlooked when a diagnosis is being made, it becomes almost impossible to detect this type of tumor.

In this study, we report about a 28-year-old woman with lymph node metastasis as the initial symptom of a germ cell tumor. We also discuss certain histological and clinical features and therapeutic options.

## 2. Patient information

A 28-year-old woman complained of multiple painless masses in the left submandibular region. The patient is in good health and does not smoke, no fixed occupation. She is married and her husband does not smoke. She has a good living environment and no family history of lymphoma or other malignant tumors. The patient underwent a series of tests prior to admission. Several laboratory data, including the negative result of a tuberculin test and the levels of beta human chorionic gonadotropin (β-HCG), lactate dehydrogenase (LDH), carbohydrate antigen 125 (CA-125), carcinoembryonic antigen (CEA), carbohydrate antigen 19-9 (CA19-9), and alpha-fetoprotein (AFP), revealed no abnormalities. Chest and abdominal CT scans showed no abnormalities. On physical examination, enlarged lymph nodes about 1 to 2 cm below the left jaw could be palpated without tenderness. Ultrasonography of the left submandibular region showed that the masses were actually a few enlarged lymph nodes. Because treatment with antibiotics was ineffective and the patient did not have other inflammatory symptoms, it was highly suspected that the enlarged lymph nodes were the site of a primary tumor, such as lymphoma, or were involved by metastatic tumor cells.

## 3. Diagnostic assessment

**Figure 1.**
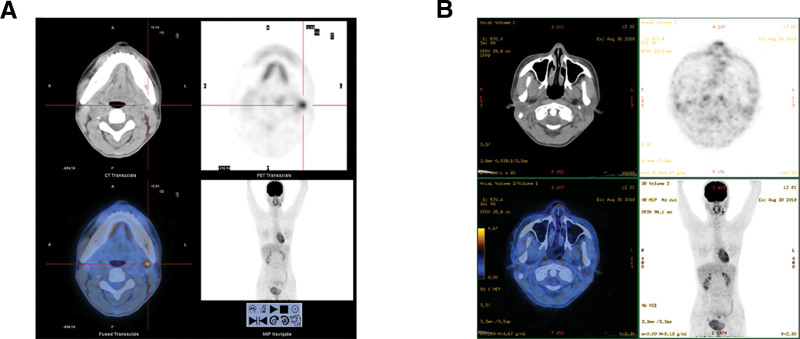
Images of whole-body ^18^F-FDG positron emission tomography-computed tomography performed during the initial clinic visit. (A) Enlarged submandibular lymph nodes were identified (the maximum diameter was about 1.2 cm). (B) No primary tumor was found in the body, especially in the nasal cavity.

With the patient’s consent, a noninvasive whole-body 18F-fluorodeoxyglucose positron emission tomography-computed tomography (^18^F-FDG PET/CT) was performed. PET/CT was performed left submandibular soft tissue nodules were identified showing increased metabolism, with the largest diameter at about 1.2 cm and the maximum SUV value at around 6.10. Multiple small lymph nodes were also identified on both sides of the neck (Fig. 1A and B). No primary tumor was found.

The excision of lymph nodes for pathological analysis was necessary to confirm the diagnosis. The Pathological biopsy of lymph node verified the occurrence of tumor metastasis and through a morphological and immunohistochemical analysis of the partial lymphadenectomy specimen, revealed that the tumor cells may have originated from a germ cell tumor.

**Figure 2.**
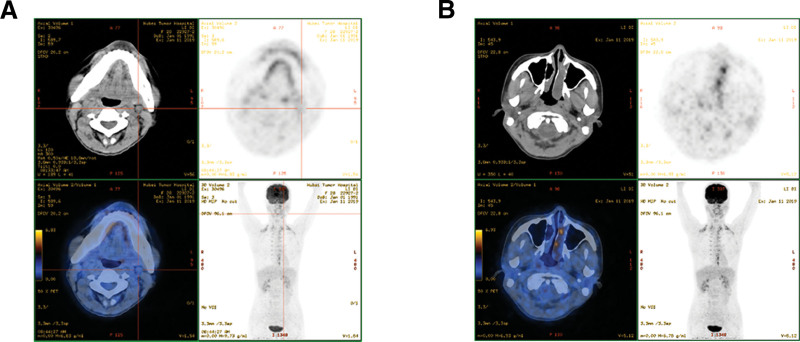
Images of whole-body positron emission tomography-computed tomography (PET-CT) performed after partial lymphadenectomy. (A) In contrast to images of the first PET-CT, the images of the second PET-CT showed that the residual submandibular lymph nodes had reduced in size (the maximum diameter was about 0.8 cm). (B) A new concealed mass emerged in the left nasal cavity.

**Figure 3.**
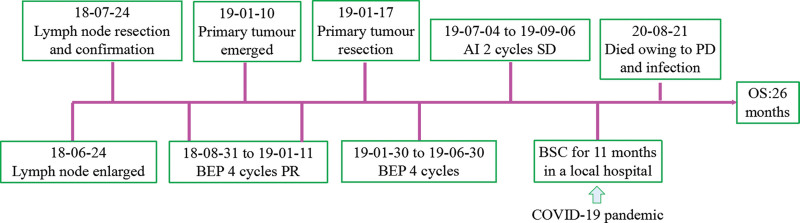
Timeline of the clinic therapy of this case report. AI = doxorubicin and ifosfamide; BEP = bleomycin, etoposide, and cisplatin; BSC = best supportive care; PD = progressive disease; SD = stable disease.

The patient received four cycles of bleomycin, etoposide, and cisplatin (BEP) chemotherapy within ~5 months. During the treatment, the residual enlarged lymph nodes became smaller, but 1 month later, the patient had progressive nasal obstruction and rhinorrhoea. Laboratory data indicated that the levels of β-HCG, LDH, CA-125, CEA, CA19-9, and AFP were normal. However, in contrast with the result of the first PET-CT, a second PET-CT revealed that although the residual submandibular lymph nodes had decreased in size, a concealed mass had emerged in the left nasal cavity (Fig. 2A and B). After complete resection was performed, it was confirmed that this primary tumor was an immature teratoma. After resection of the nasal tumor, the patient received 5 cycles of BEP chemotherapy and 2 cycles of doxorubicin and ifosfamide (AI) chemotherapy. The patient was well tolerated by chemotherapy, evaluation with no signs of relapse and no change in the sizes of the residual submandibular lymph nodes. Unfortunately, the patient died 11 months later from a lung infection and respiratory failure during the Novel Coronavirus pneumonia epidemic. The clinical therapy of this case report is shown in Figure 3.

### 3.1. Gross and microscopic pathologic findings

**Figure 4.**
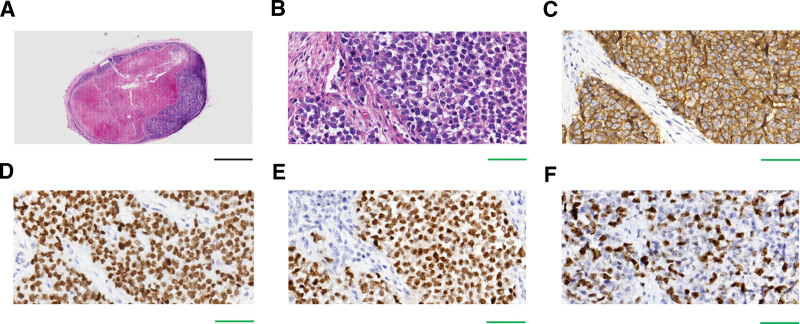
Morphological characteristics and immunohistochemical staining of metastatic tumor cells in submandibular lymph nodes. (A) The residual tumor cells were present along the marginal sinus of the lymph nodes. Extensive necrosis was evident in the centre of a lymph node. (B) The nuclei of the tumor cells exhibited round or oval contours and prominent nucleoli. Mitoses were common. (C–E) The tumor cells demonstrated membranous staining for CD56 (C) and nuclear staining for Salk 4 (D) and Oct-3/4 (E). (F) The Ki67 proliferation index appeared to be about 20% (black scale: 5 μm; green scale: 50 μm).

The lymph nodes were intact and measured 2 × 2 × 1 cm and 3 × 2 × 1.5 cm. The cut surface was homogeneous, and locally, it was light-tan to white in color. Central necrosis was observed in one lymph node. On microscopic evaluation, we found that the basic architecture of the two lymph nodes had been destroyed by the infiltration of neoplastic cells. Easily differentiated from normal lymphocytes, the metastatic tumor cells had spread in sheets or nests, invading along the marginal sinus of the lymph nodes. In the center of the two lymph nodes, extensive necrosis was evident under microscope (Fig. 4A). The polygonal or round tumor cells had distinct cell membranes and abundant granular eosinophilic cytoplasm. A majority of these cells had uniformly medium-sized nuclei with vesicular chromatin and prominent nucleoli. The mitotic rate was between 4 and 6 mitoses per 10 high-power fields (Fig. 4B). All metastatic tumor cells were diffusely positive for cluster of differentiation (CD) 56 (CD56), Sal-like protein 4 (SALL4), octamer-binding transcription factor-3/4 (OCT-3/4) (Fig. 4C–E), and vimentin. The tumor cells were negative for the expression of pan-cytokeratin (PCK), cytokeratin (CK) 5/6 (CK5/6), P40, CK7, CK8/18, epithelial membrane antigen (EMA), thyroid transcription factor 1 (TTF-1), synaptophysin (Syn), neuron-specific enolase, AFP, glypican-3, CD30, placental alkaline phosphatase (PLAP), CD117, D2-40, melan-A, human melanoma black-45 (HMB-45), S-100, sex-determining-region-Y-box transcription factor 10 (SOX10), CD99, paired box transcription factor 8, desmin, alpha-smooth muscle actin, leucocyte common antigen (LCA), CD3, CD20, anaplastic lymphoma kinase (ALK), and CD138. The samples showed a Ki-67 nuclear proliferation index of about 20% (Fig. 4F).

**Figure 5.**
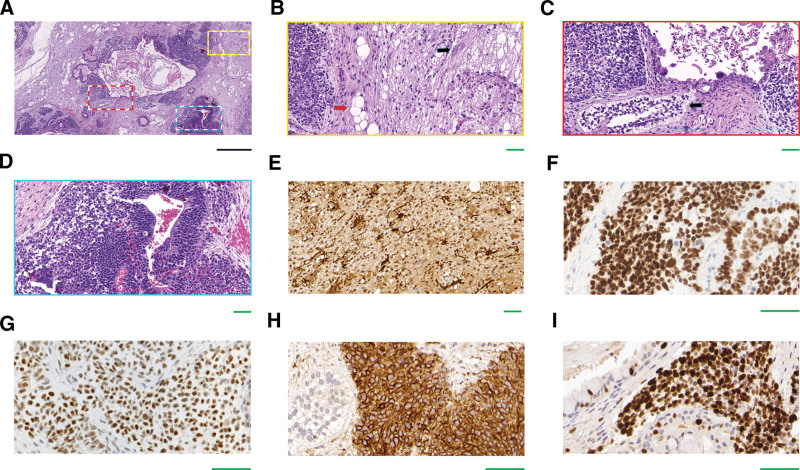
Immature teratoma in the nasal cavity. (A) Mature squamous epithelium, respiratory/bronchial epithelium, glia, adipose tissue, and smooth muscle were seen. (B) Magnification of the area marked by the yellow box in Figure 4A revealed the presence of immature neuroectoderm cells (left), mature adipose tissue (red arrow), and glia (black arrow). (C) Magnification of the area marked by the red box in Figure 4A revealed the presence of immature respiratory epithelium (black arrow). (D) Magnification of the area marked by the cyan box in (A) revealed the presence of immature neuroectodermal tissue that consisted of rosettes composed of primitive cells with increased nucleoplasmic ratios and hyperchromatic nuclei. (E–G and I) All of the immature neuroectoderm cells demonstrated membranous staining for CD56 (E), nuclear staining for Salk4 (F) and Oct-3/4 (G), and a high Ki67 proliferation index (of about 90%) (I). (H) Local glial cells demonstrated cytoplasmic staining for glial fibrillary acidic protein (black scale: 500 μm; green scale: 50 μm).

Grossly, the neoplasm from the nasal cavity measured 3.5 × 3 × 2 cm, and it was partly encapsulated. The cut surface was predominantly solid and fleshy greyish-tan in color, interspersed with cysts. The entire neoplasm was thoroughly examined under a microscope using slides stained with haematoxylin and eosin. Heterogenous components revealed that the tumor cells had differentiated along ectodermal, endodermal, and mesodermal lines, with mature and immature elements. Islands of well-differentiated glands, tubular and acinar areas, keratinised squamous epithelium and other adnexal structures, brain tissue, few bundles of smooth muscle, adipose tissue, and some areas of the respiratory/bronchial epithelium were present (Fig. 5A and B). Immature tissue of the foetal respiratory epithelium (Fig. 5C), neuroectodermal tubules and rosettes (Fig. 5D), and sheets of immature neural cells were also identified. Some tumor cells had invaded the nasal submucosa with a nested pattern of invasion. Consistent with the metastatic tumor cells, the immature neuroectodermal tubules and rosettes in the slide were also diffusely and strongly positive for CD56, SALL4, and OCT-3/4 (Fig. 5E–G).

## 4. Discussion and Conclusions

Immature teratomas that occur in the head and neck, especially those that are primarily present in the nasal cavity, are very rare. The present case concerns an extremely rare scenario, which demonstrates that submandibular lymph node metastasis can be the only and initial symptom in a patient in whom a primary tumor is absent.

In this case, confirming where the metastatic tumor cells in the lymph nodes originated from was significant. Although malignant germ cell metastasis in submandibular lymph nodes is rare, there are still some clues that point to its occurrence. For tumor cells in lymph nodes that have vesicular chromatin and prominent nucleoli, it should first be determined whether they are cells from a primary tumor or cells associated with metastasis. In adults, if tumor cells are from a primary tumor, malignant lymphoma is usually the first consideration. On the other hand, among young people, with respect to metastatic cells, carcinoma is uncommon, but the metastasis of malignant melanomas, germ cell tumors, and some special types of sarcomas should be considered among the differential diagnoses.

In lymphoma, malignant tumor cells are usually represented by the diffusive invasion and destruction of a lymph node. In the present case, the tumor cells grew in nests or sheets and were negative for a panel of immunomarkers, including LCA, CD3, CD20, CD138, and ALK. With respect to metastasis, corresponding to the characteristics of tumor-cell histology, PCK, EMA, CK7, CK8/18, CK5/6, melan-A, HMB-45, S-100, and SOX10 expression was found to be absent, which indicated that the patient did not have metastatic carcinoma or melanoma. In a previous study, the strong expression of SALL4 in tumors was identified in 18 out of 19 cases of extragonadal germ cell tumors, with a SALL4 specificity of 100%^[6]^; and the co-expression of OCT-3/4 and D2-40 further confirmed the diagnosis of the germ cell tumor.^[7]^ Thus, in the current case, the identification of the expression of SALL4 and co-expression of OCT-3/4 and D2-40 confirmed the diagnosis of the germ cell tumor. We also used additional conventional markers to confirm the diagnosis, including CD30, PLAP, CD117, glypican-3, and AFP, but the expression of these markers was negative. Because of the lack of proof for the presence of a primary tumor, the patient only received standard chemotherapy.

Immature teratomas with classical mature elements co-exist with various immature tissues that have corresponding histological characteristics such as neuroectodermal tubules and rosettes, foetal respiratory epithelium, and sheets of immature neural cells. Distinguishing between such tumor cells is usually not a complicated task. In this case, using a three-tiered grading system, we determined that the immature teratoma, with a large amount of immature neuroepithelial tissue that occupied six low-power fields in one slide, was a grade 3 tumor, according to the World Health Organization grading system. It is widely accepted that the administration of chemotherapy improves the prognosis associated with immature teratomas, and the stage and grade of a primary tumor and metastases are important predictive factors.^[8]^ However, for extragonadal immature teratomas, a radical tumor resection should always be considered for resectable masses. In addition, postoperative chemotherapy with a sufficient dose intensity of BEP was also necessary to suppress the growth of residual tumor cells.^[9]^

In this case, the patient received chemotherapy after she had undergone an operation. The patient’s condition, with respect to the inhibition of the growth of metastatic cells and the recurrence of the primary tumor, evolved favourably. However, 11 months later, she died of pulmonary infection and respiratory failure in accident.

Germ cell tumor should be considered as one of the most important differential diagnoses for adults with lymph nodes metastasis, even if a primary tumor is absent or concealed. A comprehensive pathological analysis and the detection of gene rearrangements are very important for the diagnosis of this rare disease. Radical surgery and adequate postoperative chemotherapy may not change the reality of the poor prognosis associated with this condition. Alternatively, the combination of immunotherapy associated with the inhibition of programmed cell death 1 receptor and programmed cell death ligand 1, which are immune checkpoint proteins, and anti-angiogenic therapy could be a new treatment for this disease.^[10]^ Because the patient in this case was treated 2 years ago, the drugs associated with these therapies were still rather expensive and there was little experience in cases of extragonadal germ cell tumors. Consequently, the patient did not receive the aforementioned treatments that are commonly used today. It is expected that in the future, these therapeutic approaches, including standardised operations, chemotherapy, radiotherapy, immune checkpoint inhibitor therapy, targeted gene therapy, and anti-angiogenic therapy, will be used in an increasing number of cases of extragonadal immature teratomas.

## Acknowledgments

We would like to acknowledge our collaborators from the PET center departments of Professor Dasheng Qiu and Dr LeiLi.

## Author contributions

GF and WH are responsible for collecting pathological data and writing articles. PG and XP is responsible for the overall framework of the article and writing the clinical treatment part.

XH and SH modifies the grammar of the sentence and modifies the format of the content.

Conceptualization: Guoliang Pi

Data curation: Peng Xu

Formal analysis: Peng Xu

Methodology: Guoliang Pi

Project administration: Guoliang Pi

Validation: Heshun Xia, Hongwei Shi

Writing – original draft: Fang Guo, Hongbing Wang

Writing – review & editing: Fang Guo, Heshun Xia, Hongbing Wang, Hongwei Shi
