# Lymph node metastasis as the initial symptom of a germ cell tumor in an adult: A case report: Erratum

**DOI:** 10.1097/MD.0000000000030462

**Published:** 2022-08-26

In the article, “Lymph node metastasis as the initial symptom of a germ cell tumor in an adult: A case report”,^[1]^ which published in Volume 101, Issue 30 of *Medicine*, Figures 4 and 5’s images were switched. This has been corrected.
